# Latent profiles of fatigue in inflammatory bowel disease

**DOI:** 10.1186/s12876-024-03239-2

**Published:** 2024-04-30

**Authors:** Alex Barnes, Barbara Toson, R. V. Bryant, Sutapa Mukherjee, Jane M. Andrews, Paul Spizzo, Réme Mountifield

**Affiliations:** 1 Department of Gastroenterology, Southern Adelaide Local Health Network (SALHN), Flinders Medical Centre, Flinders Drive, Bedford Park, 5042 SA Australia; 2 College of medicine and public health, Flinders University, Bedford Park, SA Australia; 3 Department of Gastroenterology, Queen Elizabeth Hospital, Woodville, SA Australia; 4 School of Medicine, Faculty of Health & Medical Sciences, University of Adelaide, Adelaide, SA Australia; 5 Adelaide Institute for Sleep Health, Flinders Health and Medical Research Institute, College of Medicine and Public Health, Flinders University, Bedford Park, SA Australia; 6 Department of Respiratory and Sleep Medicine, Southern Adelaide Local Health Network (SALHN) Flinders Medical Centre, Bedford Park, SA Australia; 7 Inflammatory Bowel Disease Service, Department of Gastroenterology and Hepatology, (CAHLN) Royal Adelaide Hospital, Adelaide, SA Australia

**Keywords:** Inflammatory bowel disease, Sleep quality, Mental health, Fatigue, Colitis, Anxiety, Depression

## Abstract

**Introduction:**

Fatigue is prevalent in people with inflammatory bowel disease (IBD) and has been associated with IBD activity, sleep quality, depression, and anxiety. This study aimed to identify fatigue profiles or clusters through latent profile analysis.

**Methods:**

An online questionnaire was administered through three tertiary IBD centres, social media and through Crohn’s Colitis Australia. Fatigue was assessed via the Functional assessment of chronic illness measurement system fatigue subscale (FACIT-F), a validated assessment of fatigue and its severity. Validated measures of anxiety, depression, IBD activity and sleep quality were also included. Latent profile analysis was performed including fatigue, sleep quality, active IBD, and depression and anxiety. The relationships between profiles and IBD and demographic data were investigated.

**Results:**

In a cohort of 535 respondents, 77% were female, the median age was 41 years (range 32–52 years), and the majority had Crohn’s disease (62%). Severe fatigue was seen in 62%. Latent profile analysis identified four distinct profiles differing by fatigue score - low fatigue, at-risk profile, active IBD, and a poor mental health profile. Female gender, obesity and opioid usage were associated with higher risk of being in the active IBD and poor mental health profile. Age over 40 was associated with lower risk of being in the poor mental health profile.

**Conclusion:**

Latent profile analysis identifies four classes of fatigue in an IBD cohort with associations with specific risk factors for fatigue along with specific IBD and demographic attributes. This has implications for the classification of fatigue in IBD and treatment algorithms.

**Supplementary Information:**

The online version contains supplementary material available at 10.1186/s12876-024-03239-2.

## Introduction

Inflammatory bowel disease (IBD) is a chronic relapsing remitting immune disorder that can affect any area of the gastrointestinal tract with extra-intestinal manifestations that includes joint and skin disease. Fatigue is a common symptom in people with IBD with a systemic review and meta-analysis reporting a prevalence of 48% [1]. The pathophysiology of fatigue in IBD is poorly understood [2, 3]. Frequently reported associations with fatigue in IBD include disease activity, sleep disturbance, anxiety and, depression [4–8].

In people with fatigue, symptom clusters have been proposed [9]. For example, in patients with advanced cancer, fatigue symptom clusters have been observed including ‘sleep, drowsiness and fatigue’ [10] and ‘sleep, depression, and fatigue’ [11]. Proposed treatment algorithms for fatigue in IBD contain flow charts that consider separate causes of fatigue in isolation [12]. Other have previously sought to identify classes of fatigue trajectories in IBD populations considering fatigue, IBD activity and psychological well-being [13]. More generally, symptom clusters in IBD have been explored in a single study considering gastrointestinal and psychological symptoms [14] producing a model similar to that reported in populations with irritable bowel syndrome [15, 16]. Others have identified that there are differences in healthcare utilisation between such symptom-defined clusters [17].

This study aimed to identify fatigue profiles or clusters in people in IBD considering known associations with fatigue using latent profile analysis incorporating fatigue, IBD activity, depression, anxiety and sleep quality. It was hypothesised that similar fatigue clusters with sleep, depression and fatigue will be seen and that there may exist a fatigue cluster that is independent of IBD activity. The authors then aimed to determine associations between demographic and IBD data and latent profile membership.

## Methods

An online questionnaire was made available to people with IBD via tertiary hospital patient email lists, private gastroenterology practice email lists and social media. Individuals with a self-reported diagnosis of IBD over 18 years of age were invited to participate. Demographic data such as age and sex were recorded, along with IBD related data including disease duration and previous surgery. Ethics approval for this study was obtained from the Southern Adelaide Human Research Ethics Committee (203.20) and informed consent was obtained from all participants.

Fatigue was measured using the FACIT-F scale which is a subscale of the Functional assessment of chronic illness measurement system (FACIT). The FACIT-F subscale has been validated as a measure of fatigue in an IBD population [18]. The FACIT-F scale includes 13 questions with responses recorded on a 5-point Likert scale, with a score ranging from 0 to 52, with a lower score indicating worse fatigue. A score less than 32 indicates severe fatigue [19].

Sleep quality was measured using the Pittsburgh Sleep Quality Index (PSQI). The PSQI is a validated tool which assesses perceived sleep quality [20]. The index consists of subscales on sleep duration, sleep disturbance, sleep latency, daytime dysfunction, sleep efficiency, overall sleep quality and medications for sleep. The score ranges from 0 to 21, with a PSQI > 5 considered to represent poor sleep quality.

IBD disease activity was assessed using the Harvey Bradshaw Index (HBI) in the case of Crohn’s disease with HBI > 5 considered active disease [21]. The patient-reported version of the HBI was used in the survey, although a decision was made to maintain the general well-being and abdominal pain score similar to the physician HBI rather than using a ten-point Likert scale [22]. The Simple Clinical Colitis Activity Index (SCCAI) was used for ulcerative colitis, with an SCCAI > 5 considered active disease [23]. The patient reported form of the SCCAI was used [24] in the survey, which has been previously validated and shown to be closely concordant with physician reported SCCAI [25].

Anxiety was assessed using the generalised anxiety disorder 7-item scale (GAD-7) [26] with a score over 5 considered mild anxiety, 10–14 moderate and greater than 15 severe anxiety. The Patient Health Questionnaire 9 (PHQ-9) was used to assess depression with a score over 5 indicating mild depression, over 10 moderate depression, and over 20 severe depression [27].

### Statistical analysis

Statistical analysis was performed using Stata SE 16 (StataCorp, College Station, TX, USA). Inadequate completion of score or index led to that result not being included. For normally distributed variables mean and standard deviation (SD) were reported with comparisons made using the student t-test. For non-normally distributed variables median, and interquartile range (IQR) were reported, with comparisons made using the Mann- Whitney U test. For categorical data Pearsons χ2 test was used or Fisher’s exact test when appropriate. If any incomplete data were present the participant was excluded.

Latent profile analysis was used to determine if respondents could be divided into groups or profiles based on responses to the questionnaire used to determine fatigue scores (FACIT-F), depression (PHQ9), anxiety (GAD7), IBD activity (SCCAI > 5 or HBI > 5), and sleep quality (PSQI). Stata latent profile analysis was used to determine the latent profile models [28]. To identify profiles of fatigue a one class model was first estimated with further classes added until the model with best fit was identified. Class size from 1 to 8 was considered. Model fit was assessed on model interpretability in addition to model performance criteria such as the Bayesian information criteria and the Akaike information criterion, and the minimum class size [29]. Entropy was calculated following determination of class size. Covariates were included based on model performance and interpretability. Posterior class membership probabilities were calculated for each survey response. Each survey response was assigned to a profile based on the posterior class membership probabilities. Multinomial regression was undertaken to assess for predictors of class membership.

## Results

There were 670 responses to the online questionnaire, following exclusions for any incomplete data there were 535 responses (79.8%) included in the analysis (see Table 1). Median age was 41 years (32–52), with most being female (77%), the majority had Crohn’s disease (61%). The mean disease duration was 10 years (5–19), 32% had undergone surgery for IBD and around half were on biologics (53%) (see Table 1).

**Table 1:** Cohort demographics and inflammatory bowel disease (IBD) data, Severe fatigue defined by FACIT-F < 32, clinically significant anxiety defined by GAD-7 > 10, clinically significant depression defined by PHQ-9 > 15

IBD and demographic data	
n	535
Gender (% female)	77.4
Age (median (IQR))	41 (32–52)
Crohn’s disease (%)	61.3
IBD years diagnosed (median (IQR))	10 (5–19)
IBD related surgery (%)	32.5
Obesity (%)	36.2
Active smoking (%)	6.8
Corticosteroids (%)	9.04
Aminosalicyate (%)	33.2
Biologics (%)	52.8
Immunomodulators (%)	37.04
Opioids (%)	14.8
Medications for sleep (%)	13.6
Colecalciferol (%)	28.0
Harvey Bradshaw index (mean(SD))	7.1 (3.2)
Simple clinical colitis activity index (mean(SD))	7.2 (2.8)
Clinically significant anxiety (%)	32.8
Clinically significant depression (%)	20.8
Severe fatigue (%)	57

Latent profile analysis was undertaken including fatigue scores (FACIT-F score inverted), depression scores (PHQ9), anxiety scores (GAD7), sleep quality (PSQI) and IBD activity (SCCAI > 5, HBI > 5). Covariates were included in the model such as age, IBD subtype and BMI over 25. A four-profile solution based was chosen (see supplementary Table 1, entropy was adequate at 0.82).

**Fig. 1:**
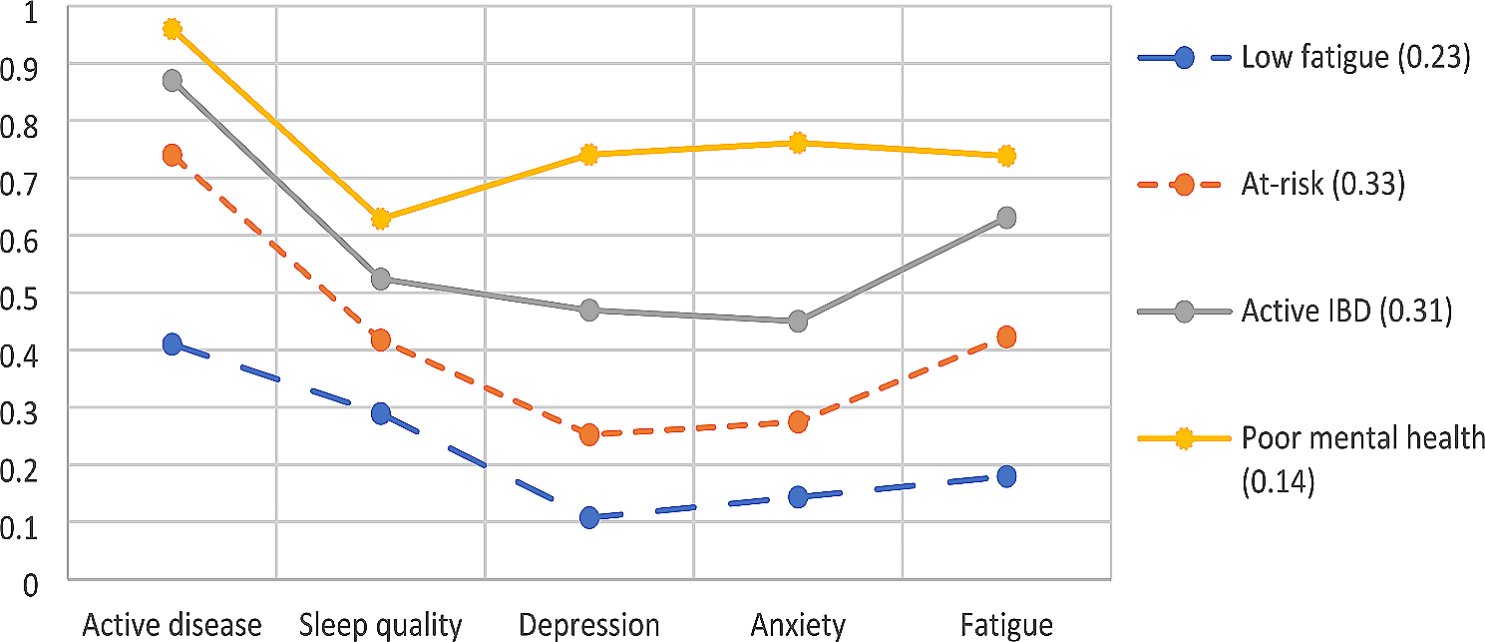
Latent profiles of determinants of fatigue. Figure illustrates the characteristics of each profile based on reported anxiety, depression, IBD activity, sleep quality and fatigue levels. A minority were in the poor mental health profile (14%), with the majority in the at risk profile (33%), with similar proportion in the active IBD profile (33%). Scores have been normalised by highest possible response for each score

The latent profiles (See Fig. 1; Table 2) were named as follows: the low fatigue profile (23%) – encompassing mild levels of fatigue and low levels of depression and anxiety; the poor mental health profile representing the smallest group (14%) characterised by severe anxiety and depression; the active IBD profile (31%) with high levels of IBD activity and associated poor sleep quality, but only mild-moderate mental health impairment. Finally, there was the at-risk profile (33%), being the largest profile, with mild levels of depression and anxiety, and moderate levels of fatigue.

**Table 2:** Mean values in each latent profile – with interpretation based on established cut offs. IBD (inflammatory bowel disease) activity refers to the proportion with clinically active IBD. Sleep quality via the Pittsburgh Sleep Quality Index. Depression via Patient Health Questionnaire 9 scoring. Anxiety via the Generalised anxiety disorder − 7 score. Fatigue by the Functional assessment of chronic illness measurement system fatigue score

Profile	Low fatigue	At-risk	Active IBD	Poor mental health
IBD activity	0.41	0.74	0.87	0.96
Sleep quality	6.07	8.76	11	13.2
Depression	Nil	Mild	Moderate	Severe
Anxiety	Nil	Mild	Moderate	Severe
Fatigue	Mild	Moderate	Severe	Severe

**Fig. 2:**
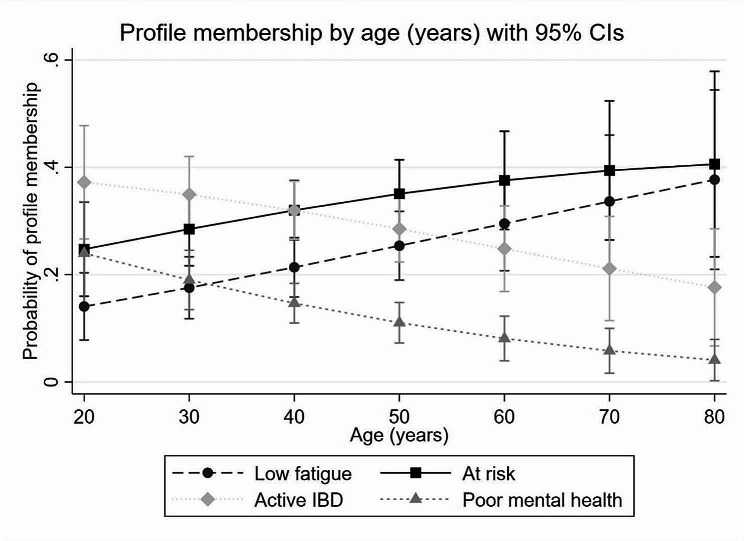
Latent profiles of determinants of fatigue. Age (covariate in latent model) plotted against each latent profile – low fatigue, at risk, active IBD, poor mental health

As age increased there was a decreased probability of measurement of the higher fatigue and mental health profiles and decreased probability of membership in the lower fatigue profiles (see covariate plotting in Fig. 2). No significant change in profile membership was seen with IBD subtype (see supplementary Fig. 1).

Female gender, opioid usage and obesity were associated with membership of higher fatigue profiles (multinomial regression with low fatigue profile as base see Table 3). Age over 40 was associated with decreased likelihood of membership in the poor mental health profile. Current smoking status was associated with increased likelihood of being in the poor mental health profile and the at-risk profile but not in the active IBD profile. Corticosteroid usage was associated with increased likelihood of membership in the poor mental health class. No differences were seen with IBD subtype, IBD duration, or any biologic or immunomodulator usage.

**Table 3:** Multinomial regression analyses with relative risk ratio reported relative to low fatigue profile. IBD (inflammatory bowel disease)

Profile	At-risk	Active IBD	Poor mental health
Age over 40	1.00 (0.63–1.60) *p* = 0.99	0.63 (0.39-1.00) *p* = 0.051	**0.49 (0.27–0.88) ** ***p*** ** = 0.017**
Age over 60	1.19 (0.64–2.24) *p* = 0.57	0.84 (0.43–1.63) *p* = 0.60	0.39 (0.14–1.2) *p* = 0.08
Female gender	**2.16 (1.30–3.61) ** ***p*** ** = 0.003**	**2.84 (1.65–4.87) ** ***p*** ** < 0.001**	**2.72 (1.38–5.39) ** ***p*** ** = 0.004**
Obesity	**1.80 (1.04–3.13) ** ***p*** ** = 0.037**	**3.32 (1.93–5.71) ** ***p*** ** < 0.001**	**2.65 (1.39–5.05) ** ***p*** ** = 0.003**
Ulcerative colitis	1.04 (0.65–1.67) *p* = 0.86	1.03 (0.64–1.67) *p* = 0.90	1.01 (0.56–1.82) *p* = 0.97
Crohn’s disease	0.78 (0.49–1.26) *p* = 0.32	0.87 (0.54–1.41) *p* = 0.58	0.88 (0.49–1.59) *p* = 0.68
Corticosteroids	2.49 (0.88–6.99) *p* = 0.083	2.65 (0.94–7.3) *p* = 0.065	**4.12 (1.37–12.39) ** ***p*** ** = 0.012**
Opioids	**2.77 (1.15–6.66) ** ***p*** ** = 0.023**	**4.18 (1.78–9.83) ** ***p*** ** = 0.001**	**4.21 (1.63–10.89) ** ***p*** ** = 0.003**
Aminosalicyate	1.49 (0.90–2.48) *p* = 0.12	1.24 (0.74–2.01) *p* = 0.41	1.34 (0.72–2.49) *p* = 0.36
Immunomodulators	0.88 (0.54–1.43) *p* = 0.61	1.34 (0.83–2.18) *p* = 0.23	0.97 (0.53–1.78) *p* = 0.94
Biologics	0.91 (0.57–1.45) *p* = 0.70	0.98 (0.61–1.56) *p* = 0.93	0.83 (0.47–1.48) *p* = 0.53
Vitamin D	1.35 (0.80–2.29) *p* = 0.26	1.08 (0.63–1.86) *p* = 0.78	1.78 (0.95–3.33) *p* = 0.070
Previous IBD surgery	1.09 (0.67–1.77) *p* = 0.71	0.86 (0.52–1.41) *p* = 0.55	0.79 (0.43–1.47) *p* = 0.46
IBD over 10 years since diagnosis	1.57 (0.98–2.50) *p* = 0.056	0.80 (0.50–1.29) *p* = 0.36	0.96 (0.54–1.70) *p* = 0.88
Current smoking	**3.65 (1.02–12.98) ** ***p*** ** = 0.046**	2.98 (0.81–10.92) *p* = 0.099	**4.86 (1.25–18.91) ** ***p*** ** = 0.023**

## Discussion

For the first time in the IBD literature this study used latent profile analysis to distinguish four fatigue profiles, differing by sleep quality, IBD activity, depression, and anxiety. The higher fatigue profiles were associated with opioid usage, younger age, female gender, corticosteroid usage and obesity. Depression and anxiety were closed related across the different profiles, similarly IBD activity and sleep quality remained related across the different profiles. The profile with the highest fatigue scores saw poor sleep, IBD activity and depression present in at least moderate severity.

The importance of mental conditions was highlighted by this data with moderate-high levels of depression and anxiety seen in the class with a high probability of severe fatigue. This may in part be a physiological consequence of the neurological effects of active IBD associated inflammatory cytokines [30, 31]. There are likely bidirectional relationships between fatigue and mental health conditions, and mental conditions and IBD activity [32, 33] making causation difficult to assess. Sleep disturbance has also been associated with worse depression or anxiety.

There was a profile referred to as ‘active IBD’ that had high proportion of active IBD and poor sleep quality with low-moderate anxiety/depression scores. Clinically active IBD certainly influences sleep quality and perhaps addressing IBD activity in those in this profile will lead to improvement in both aspects and reduce the likelihood of severe fatigue. Our initial hypothesis was incorrect – there was no profile with significant levels of fatigue and low IBD activity. IBD activity in a way mirrored fatigue scores. It is important to note here that this is clinical IBD activity rather than objective IBD activity (calprotectin/endoscopy based), and consequently may relate to IBS related symptoms that are common in people with inactive IBD [34]. These IBS-like symptoms can often be influenced by other factors such as depression or anxiety.

Females were more likely to be in the higher fatigue and mental health profiles. Fatigue is more commonly seen in females [35] although in IBD populations gender differences in fatigue have been mixed [1]. Similarly, depression and anxiety are more common in females [36–38] which perhaps explains the observed associations with the profiles seen here. Variance in profile membership was seen with age but not with IBD duration.

Corticosteroid usage was more common in the poor mental health class. This may relate to the medications influence on mental health and to its usage – generally in those with clinically active IBD. The association between corticosteroids and high levels of fatigue may be due to its association with clinically active IBD. Opioid usage, and in particular opioid misuse, has been related to fatigued, perhaps due to associated sedation and have also been associated with more severe IBD [39–41].

The reported causes of fatigue in IBD are many and varied with current approaches suggesting considering causes in isolation with approaches varying from considering causes sequentially or in parallel [2, 12]. The data here suggests that the common causes of fatigue frequently coexist – for example IBD activity and sleep were closely related. The authors would suggest that those presenting to IBD clinic with severe fatigue be screened for depression and evaluated for active IBD before pursuing other possible aetiologies.

Limitations of this study include selection bias a result of the use of an online questionnaire that may attract people with fatigue or sleep problems. Similarly, the form of survey and method of recruitment is likely responsible for the predominantly female cohort. The proportion of participants with Crohn’s disease was above that present in Australian prevalence data [42]. Reporting bias may also be significant, noting a study of people with Crohn’s disease reported worse sleep quality than that observed by objective measures [43]. Data on other medical conditions study participants may have that may influence fatigue, such as heart failure, was not available. There is no gold standard measure for choosing a latent profile measure – here we used statistical measures of model performance along with model interpretability and relevance to the previous literature.

The absence of an objective measure of IBD activity is also considered a limitation. A more valid approach would be to incorporate measures such as faecal calprotectin or endoscopic activity to define objective disease activity in addition to patient reported disease activity. Understanding the associations between fatigue profile membership and objective and subjective IBD activity would be valuable. Similarly, the inclusion of socioeconomic data in the model or subsequent analysis may also be valuable. Given the nature of data collection there was no opportunity to assess for anaemia that been associated with fatigue [6]. However, as others have noted [1] anaemia was not associated with fatigue in numerous cross-sectional studies, and hence its lack of inclusion in the model here is not considered a significant limitation [44–46].

Reviewing the plot of the latent profiles (see Fig. 2) one may see that the ‘low fatigue’ and ‘at risk’ profiles are in some areas parallel – suggest that this may represent different severities of the same profile referred to as the Salsa effect [47]. However, the authors would note that the ‘at risk’ profile has a sharper rise in IBD activity and fatigue – suggesting that perhaps the increase in IBD activity leads to greater fatigue and a comparatively smaller increase in anxiety, depression, and sleep quality scores – and would argue that this does not represent simply the ‘low fatigue’ profile at a greater severity.

It would be valuable to assess how fatigue profiles change over time, alongside influencing factors and the prognostic relevance of fatigue on IBD outcomes. Current evidence suggests that fatigue remains stable in the majority of IBD patients over time [13]. The latent profiles of fatigue defined in this study add granularity to factors associated with fatigue in IBD patients, adding further opportunities to address these debilitating and prevalent symptoms.

## Conclusions

Latent profile analysis identified four profiles differentiated by levels of fatigue. The observed profiles suggest that the common risk factors for fatigue in IBD will typically co-exist. The association between depression and fatigue underlines the importance of screening for depression during IBD clinic. Attention should also be given to other factors associated with higher fatigue profiles such as obesity, opioid usage and corticosteroid usage. Further research should consider changes in fatigue profiles over time.

### Electronic supplementary material

Below is the link to the electronic supplementary material.

Supplementary Material 1

## Data Availability

The data underlying this article are available upon request to Dr Alex Barnes at alex.barnes@sa.gov.au.
