# Supplementary material for: Latent profiles of fatigue in inflammatory bowel disease
Source: BMC Gastroenterol. 2024 Apr 30;24:148. doi: 10.1186/s12876-024-03239-2 (PMC11061964; doi:10.1186/s12876-024-03239-2)

Supplementary Table 1: Latent class profile model statistics for different number of profiles. AIC – alkaline information criterion, BIC – Bayesian information criteria, LL – log-likelihood. Df – degrees of freedom.

| Profiles | LL | df | AIC | BIC |
| --- | --- | --- | --- | --- |
| 1 | -7291.11 | 9 | 14540.21 | 14578.69 |
| 2 | -6836.24 | 19 | 13710.18 | 13791.7 |
| 3 | -6700.46 | 29 | 13458.91 | 13582.88 |
| 4 | -6641.87 | 39 | 13361.73 | 13528.45 |
| 5 | -6613.66 | 49 | 13325.32 | 13534.78 |
| 6 | -6596.68 | 59 | 13311.36 | 13563.58 |
| 7 | -6580.26 | 67 | 13298.52 | 13593.48 |
| 8 | -6551.06 | 79 | 13260.11 | 13597.82 |

Supplementary figure 1: Latent profiles of determinants of fatigue. Male gender (covariate in latent model) plotted against each latent profile – low fatigue, at risk, active IBD, poor mental health.


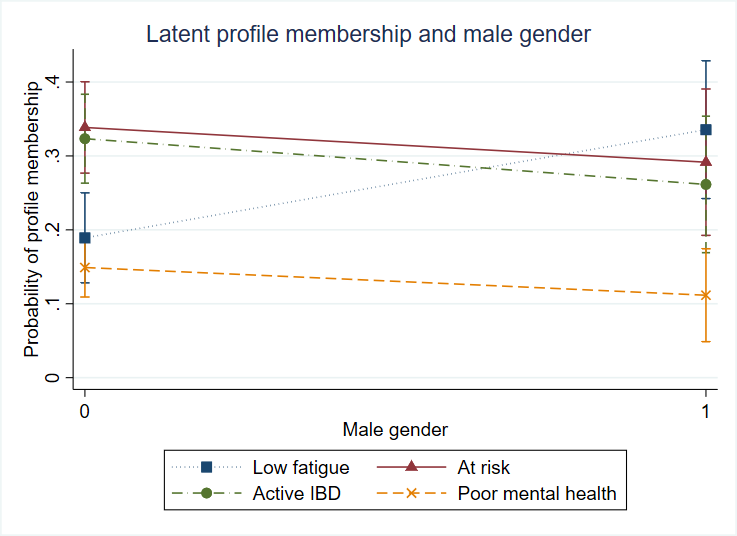


Supplementary figure 2: Latent profiles of determinants of fatigue. Body mass index over twenty five (covariate in latent model) plotted against each latent profile – low fatigue, at risk, active IBD, poor mental health.


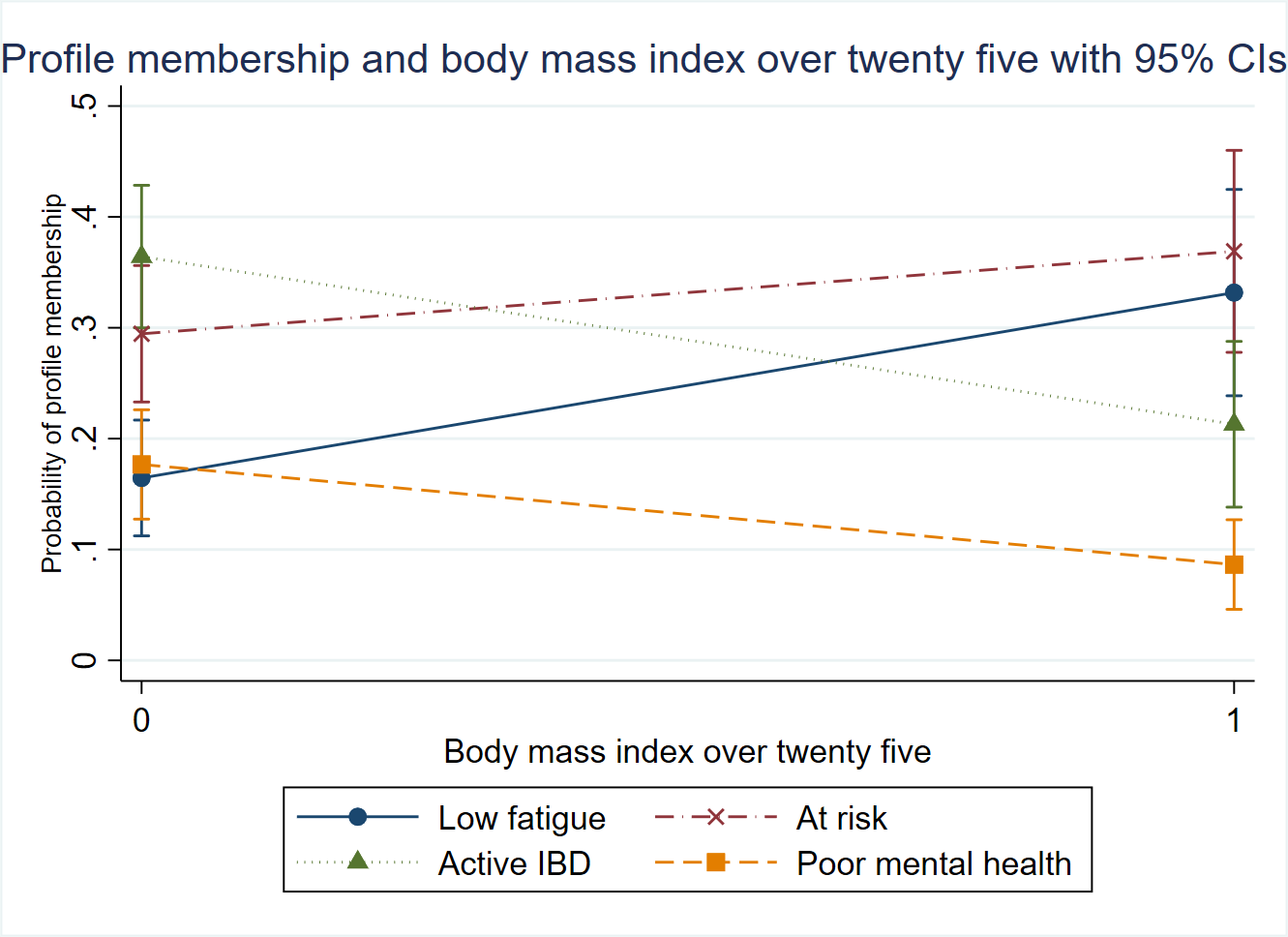


Supplementary figure 3: Latent profiles of determinants of fatigue. Crohn’s disease (covariate in latent model) plotted against each latent profile – low fatigue, at risk, active IBD, poor mental health.


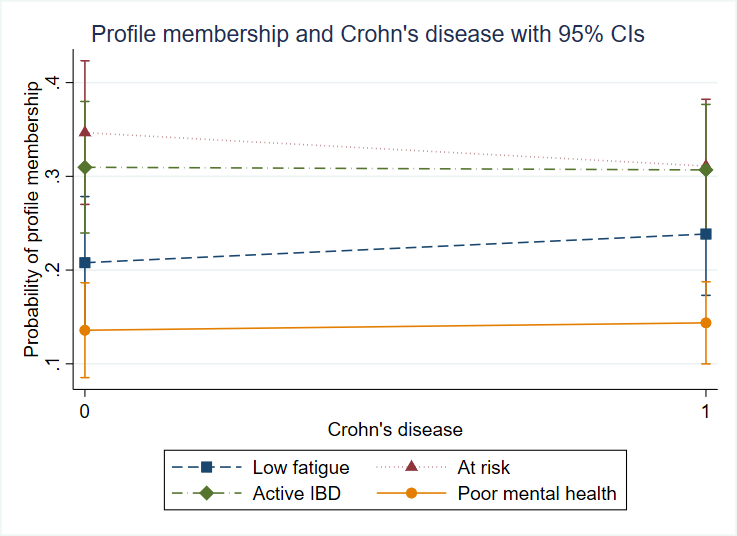

Supplement: Supplementary file 1 — Supplementary Material 1 [file 12876_2024_3239_MOESM1_ESM.docx]
